# Interdependence of social-ecological-technological systems in Phoenix, Arizona: consequences of an extreme precipitation event

**DOI:** 10.1186/s43065-023-00085-6

**Published:** 2023-08-18

**Authors:** Alysha Helmrich, Amanda Kuhn, Anaís Roque, Ameyalli Santibanez, Yeowon Kim, Nancy B. Grimm, Mikhail Chester

**Affiliations:** 1 College of Engineering, University of Georgia, Athens, GA USA; 2 School of Life Sciences, Arizona State University, Tempe, AZ USA; 3 Department of Anthropology, The Ohio State University, Columbus, OH USA; 4 School of Sustainable Engineering and the Built Environment, Arizona State University, Tempe, AZ USA; 5 Department of Civil and Environmental Engineering, Carleton University, Ottawa, ON Canada

**Keywords:** Flooding, Resilience, SETS, Critical infrastructure, Interconnected, Infrastructure systems

## Abstract

Complex adaptive systems – such as critical infrastructures (CI) – are defined by their vast, multi-level interactions and emergent behaviors, but this elaborate web of interactions often conceals relationships. For instance, CI is often reduced to technological components, ignoring that social and ecological components are also embedded, leading to unintentional consequences from disturbance events. Analysis of CI as social-ecological-technological systems (SETS) can support integrated decision-making and increase infrastructure’s capacity for resilience to climate change. We assess the impacts of an extreme precipitation event in Phoenix, AZ to identify pathways of disruption and feedback loops across SETS as presented in an illustrative causal loop diagram, developed through semi-structured interviews with researchers and practitioners and cross-validated with a literature review. The causal loop diagram consists of 19 components resulting in hundreds of feedback loops and cascading failures, with surface runoff, infiltration, and water bodies as well as power, water, and transportation infrastructures appearing to have critical roles in maintaining system services. We found that pathways of disruptions highlight potential weak spots within the system that could benefit from climate adaptation, and feedback loops may serve as potential tools to divert failure at the root cause. This method of convergence research shows potential as a useful tool to illustrate a broader perspective of urban systems and address the increasing complexity and uncertainty of the Anthropocene.

**Supplementary Information:**

The online version contains supplementary material available at 10.1186/s43065-023-00085-6.

## Introduction

Critical infrastructures (CI) are complex adaptive systems [1] operating in increasingly interconnected and complex environments [2, 3]. CI are typically defined as physical and cyber systems and assets that are essential to maintaining a functioning society, from economic security to public health [4]; however, there have been recent challenges to expand the definition beyond technological systems to include human capabilities and ecological systems [5–7]. We assert that CI exist as social, ecological, and technological systems (SETS), indicating that impacts in one component will reverberate to others [1, 8, 9]. CI are traditionally considered technological systems, but they are designed within and rely upon social and environmental processes [10]. Technological-ecological interactions include extraction, utilization, and/or transformation of resources; additionally, technological systems are designed to ‘withstand’ ecological disturbances [9, 11]. Technological-social interactions are also innately embedded in CI, as CI are developed to meet the needs and expectations of society and must abide by societal and organizational rules, regulations, and norms [9, 12, 13] established by urban policy and planning, insurance policies, demand trajectories, etc. [14, 15]. It is pertinent to examine CI as SETS to identify interconnections, dependencies, and interdependencies (hereafter, relationships).

Complexity is defined by its entangled relationships and emergent behaviors [16], but this elaborate web of interactions often conceals relationships and feedback loops, creating multiple potential pathways of disruption [1, 13, 17]. Pathways of disruption may be direct or indirect, physical or non-physical (Table 1). Cascading failures, in particular, have largely been characterized as unpredictable due to the complex nature of such events [1, 13]. These disruptions may be triggered by high-impact, low-probability or low-impact, high-probability disruptive events, and the increasing connectedness of infrastructure networks increases the likelihood of cascading failures [1, 13, 18, 19]. Despite this vulnerability, relatively few studies exist exploring CI relationships and the risk of cascading failure within the United States, and fewer consider SETS dynamics [13, 17, 20–25]. This research gap has also been identified by the emergent field of Multisector Dynamics, which identifies cascading effects and failures, path dependencies, and tipping points as focal analytical challenges to complex systems of systems [26].

**Table 1:** Pathways of disruption

**Classification**	**Characterization** [17]	**SETS Expansion**
Direct Physical	Impact to physical infrastructure	Impact to built infrastructure or natural environment, including biotic and abiotic factors
Indirect Physical^a^	Disruption resulting from other interconnected or co-located infrastructure	Disruption resulting from other interconnected or co-located infrastructure, built or natural
Direct Non-physical	Impacts on human health, behavior, and decision making	Impacts on social factors (e.g., physical health, behavior, decision-making) from individual to collective scales
Indirect Non-physical^a^	Disruption resulting from loss of information, social, financial, etc. resources	Disruption resulting from loss of intangible resources (e.g., mental health, knowledge, money)

^a^Representative of cascading failures

Climate change further increases the complexity of CI relationships [27–29]. Here, we examine the impacts of an extreme precipitation event on CI relationships in the Phoenix Metropolitan Area (hereafter, Phoenix) to identify potential pathways of disruption and potential cascading failures. Located in central Arizona, Phoenix homes nearly 4.9 million residents. Phoenix has been experiencing rapid population growth and urbanization for decades, creating one of the largest and fastest-growing metropolitan areas in the United States [30, 31]. The climate is hot and arid, with daily maximum temperatures commonly surpassing 37^▫^C from June to September and annual precipitation ranging from 127 to 203 mm [32–34]. The summer monsoon season, characterized by intense convective storms, generates a high risk of flash flooding, which may cause death, injuries, and property damage [33, 34]. Furthermore, changing climatic conditions increase demand on CI through gradual climatic shifts (stress) and extreme weather events (shocks) [12, 35, 36]. Phoenix is experiencing less frequent and more intense monsoon events compared to past trends [37]. We consider a severe monsoon event (i.e., a short, intense, and highly localized storm) to identify relationships within the urban system. We explore an abstract severe monsoon event, rather than a particular intensity, because climate change is complex and, therefore, unpredictable, meaning an estimate of a worst-case precipitation event today is unlikely to maintain worst-case status in the future [27, 36, 38–40]. Rather than focusing on increasing the robustness of technological infrastructures to a particular intensity, here we emphasize the SETS relationships and stakeholder networking necessary to create a more resilient urban system.

**Fig. 1:**
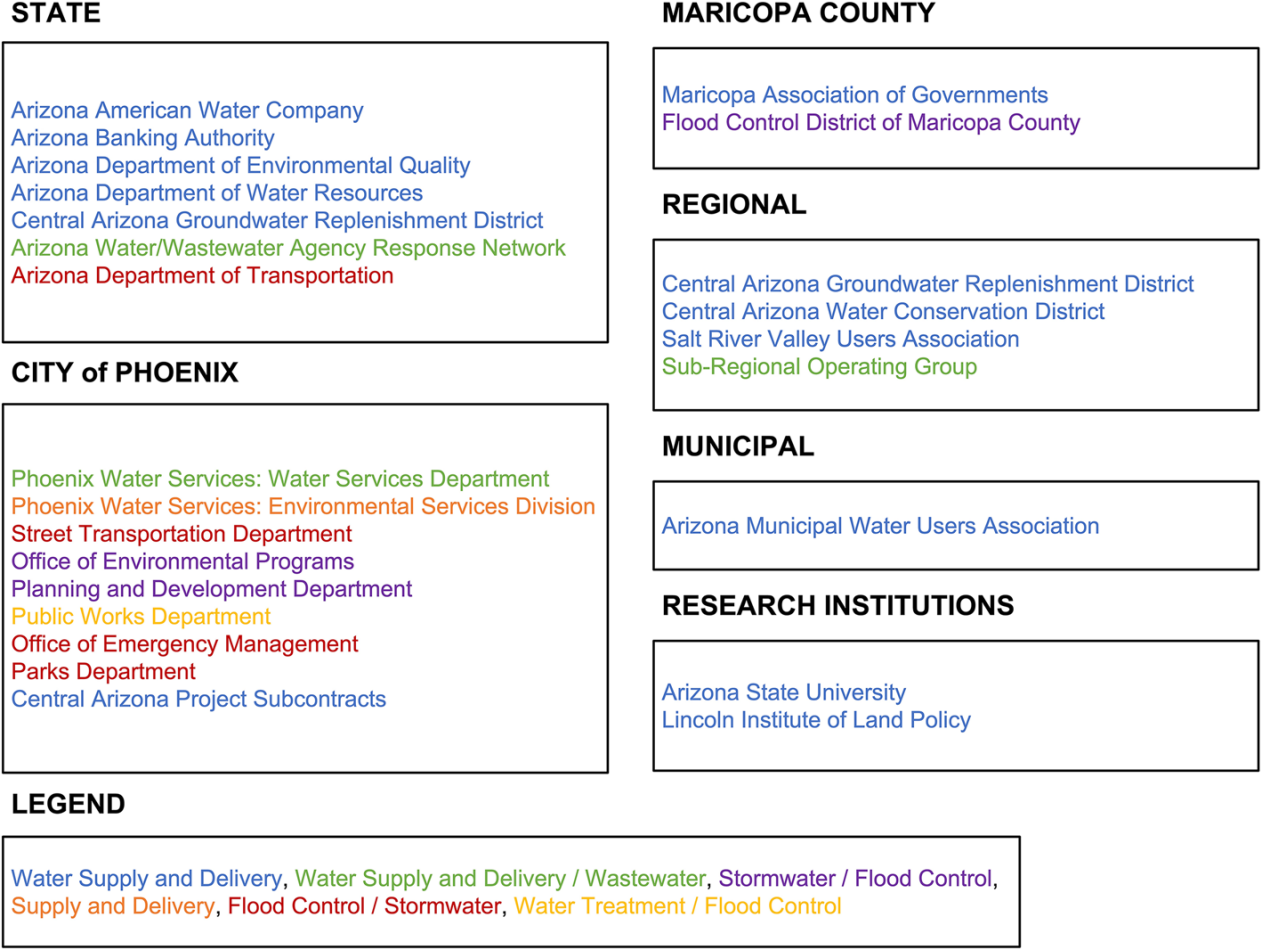
Select flood managers operating in Phoenix, AZ

Effective planning and response to changing monsoon characteristics in a complex urban system requires coordination between infrastructure managers and key stakeholders across SETS domains. The Flood Control District of Maricopa County (FCDMC) is the regional agency responsible for the development and administration of flood-management policy [41], as water management is the ‘defining activity of living in the desert’ [42]. Hydrologic processes are tightly governed across spatial and temporal scales as well as SETS sectors to address the interacting pressures of chronic drought and flood risk [43]. Phoenix approaches stormwater management with a bottom-up approach, integrating policies, public resources, and green and gray elements administered in decentralized modes [44, 45]. Therefore, monsoon planning and response managers (hereafter, flood managers) encompass an expansive group of actors who directly or indirectly affect flood mitigation, preparedness, response, and recovery. These actors include, but are not limited to, hydrologists, meteorologists, urban planners, structural engineers, transportation engineers, finance managers, environmental policy analysts, floodplain permit specialists, and public information officers (Fig. 1). This diverse group of flood managers oversees many social, ecological, and technological components, listed and described as follows:

- *Natural floodplains* provide flood water storage and conveyance and are managed for monsoon planning through floodplain management and water quality, as well as monsoon response through dam release and riverine flood response [46].
- *Green stormwater infrastructure* are designed and implemented by private and public actors across governance scales to promote in-situ stormwater infiltration, evapotranspiration, and reuse [47–49]. Private property owners are responsible for managing severe pluvial flooding [45], and socioeconomic status limits implementation of green infrastructure projects such as rain gutters and barrels, vegetation, altered yard slope [50].
- *Gray stormwater infrastructures* are human-engineered stormwater infrastructure (e.g., culverts), and they are often managed by municipalities. In Phoenix, responsible parties include the City of Phoenix Water Services Department, Street Transportation Department, Office of Environmental Programs, Planning and Development Department, and Public Works Department [32]. Similar entities exist in the other municipalities of the Phoenix metropolitan area. Green and gray stormwater infrastructure components can be used in conjunction, providing a hybrid solution.
- *Transportation infrastructures*, governed by Arizona Department of Transportation (ADOT) and, for example, the Phoenix Street Transportation Department, are planned in coordination with stormwater drainage planning to provide conveyance to structural and natural drainage corridors [32, 45, 51].
- *Community education and citizen science programs* can promote risk knowledge and awareness to generate adaptive capacity in flood-prone communities through formal and informal activities [52]. The FCDMC’s ‘Report-a-Flood’ initiative is an example of integrating citizen-based knowledge into institutional flood management [45, 53].

We posit that a SETS approach to urban flood management can better prepare water managers to respond to extreme precipitation events, where the identification of CI relationships may reveal vulnerabilities, establish cooperative data collection, and promote coordination in monsoon planning and response. We assess the impacts of an extreme precipitation event in Phoenix, Arizona (AZ) using an illustrative causal loop diagram, developed through semi-structured interviews with researchers and practitioners and cross-validated with a literature review, to address three questions:

1. What social, ecological, and technological components are impacted by extreme precipitation events within Phoenix?
2. Do the relationships between social, ecological, and technological components create potential pathways of disruption?
3. Are there SETS relationships that provide opportunities or challenges toward resilience pathways for monsoon planning and response?

The holistic vision of SETS allows us to assess opportunities for radical change within CI development [3]. We apply a SETS perspective to 1) identify interconnected, dependent, and interdependent infrastructures and 2) map potential pathways of disruption during an extreme weather event. This analysis expands upon previous work focusing on technological cascading failures to explore impacts on social and ecological components. Recognizing all three components within CI minimizes the potential to reduce the complexity of infrastructure problems, which can lead to unintentional consequences and failures [9, 23, 27, 54].

## Results

**Fig. 2:**
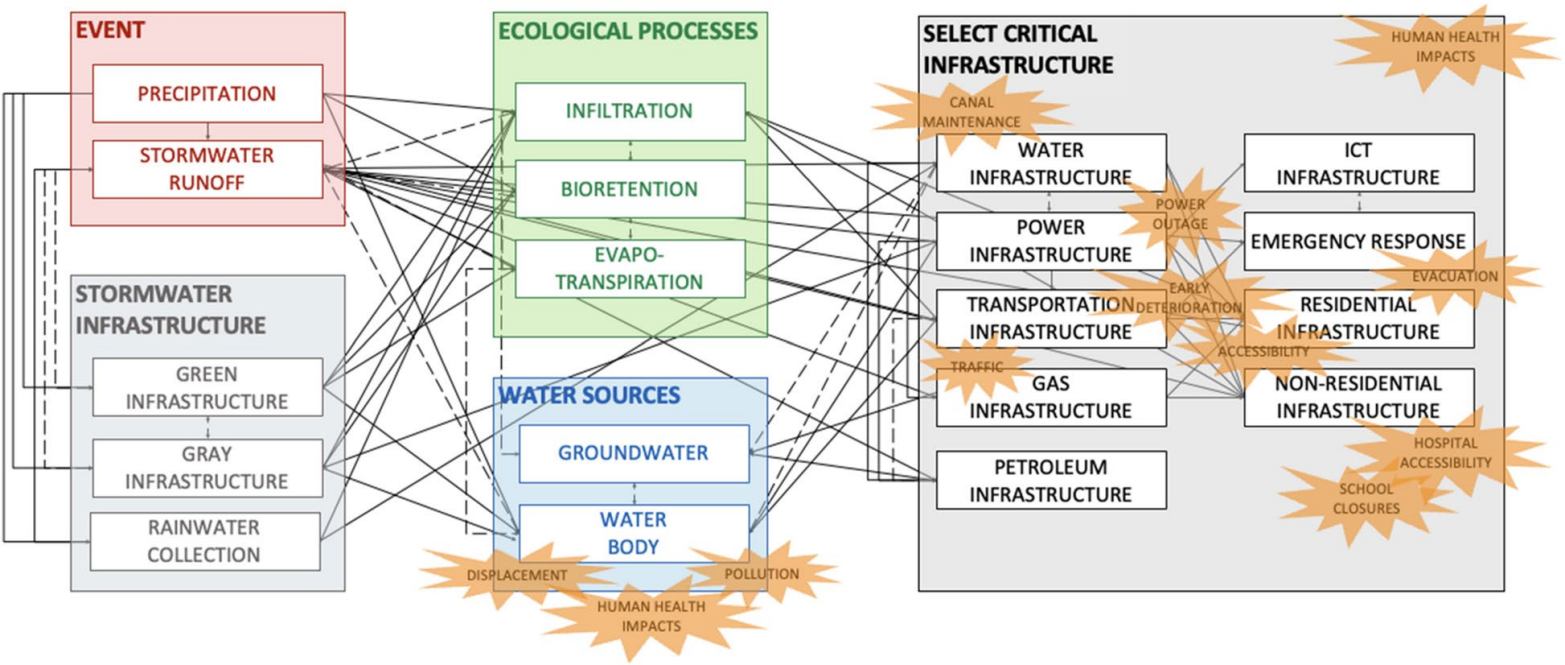
Illustrative causal loop diagram depicting interconnections between ecological and technological components with a social overlay. The 82 depicted relationships are catologued in Supplementary Information Table 1. Solid lines represent dependencies, and dashed lines represent interdependencies. This is not exhaustive, and the causal loop diagram presented does not capture every relationship. The diagram provides an overview of system behavior from a multidisciplinary perspective and encourages flood managers to consider the complexity of their system [55, 56]. It also represents a snapshot immediately following the hazard and does not capture long-term adaptations or transformations. This diagram was generated by the authors, urban resilience experts, and flood managers

A multidisciplinary approach to CI via SETS allows for numerous complex relationships to be captured that may otherwise be easily overlooked. The presented causal loop diagram (Fig. 2) consisted of 19 components and 82 relationships, with an associated 12 social impacts.

### System components

**Fig. 3:**
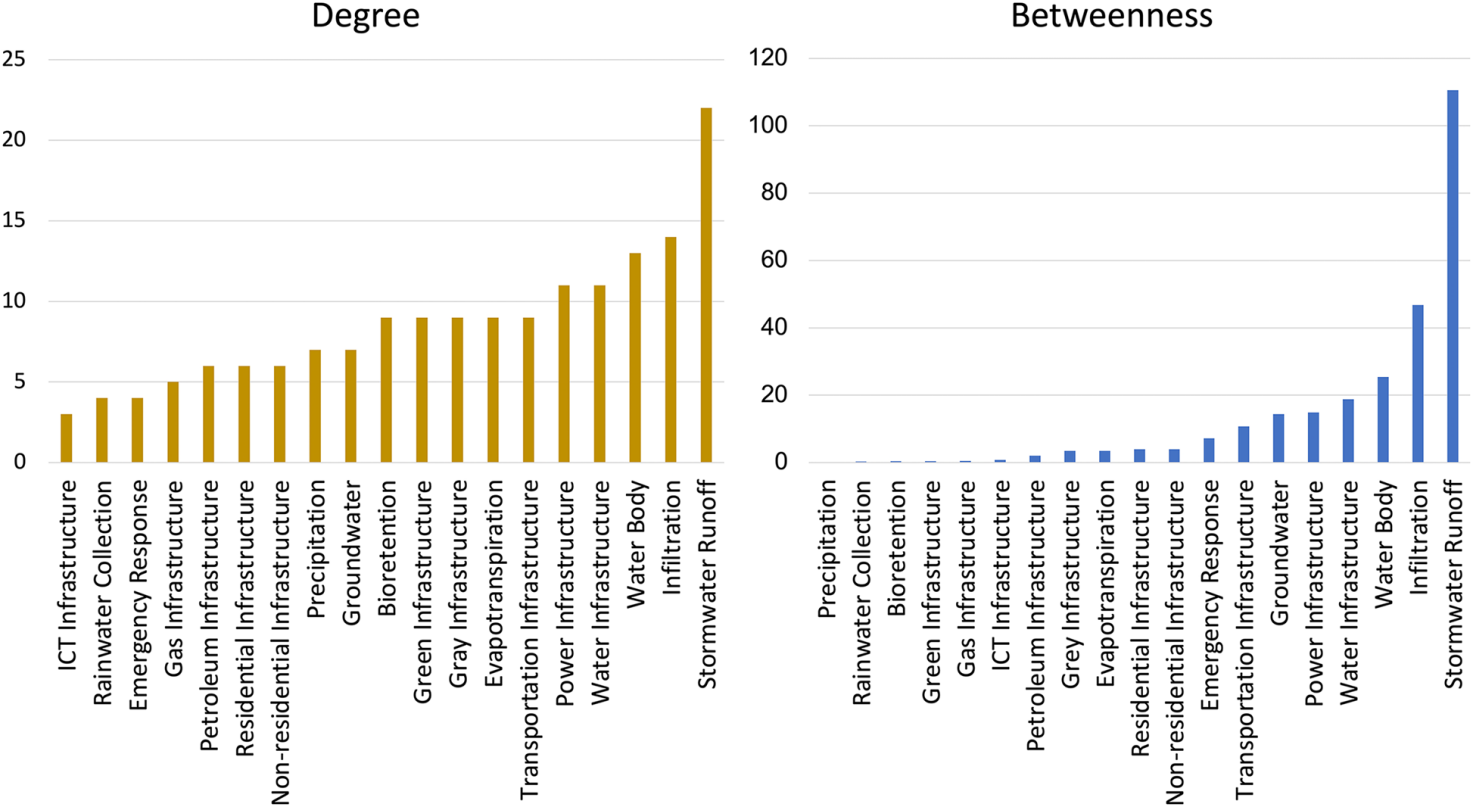
The bar chart to the left (gold bars) depicts the degree centrality of the system components. The bar chart to the right (blue bars) depicts the betweenness values of the system components

A total of 19 *system components* (depicted by rectangles in Fig. 2) were identified to represent the monsoon planning and response system. Ecological components include the monsoon event (precipitation and stormwater runoff), ecological processes (infiltration, bioretention, evapotranspiration), and water sources (groundwater and water body). Technological components consisted of potable water, power, transportation, gas, petroleum, information communication technology (ICT), residential, and non-residential infrastructures. The three stormwater infrastructure components (green infrastructure, gray infrastructure, and rainwater collection) may be classified as ecological–technological components. Finally, emergency response is a social–technological component. There are no social components, as the social component was integrated as potential failure consequences (shown as orange bubbles in Fig. 2). The social components (e.g., governance, public health, education, etc.) are embedded within the ecological and technological components and their relationships. Network analysis was used to calculate the degree centrality of each component, or the number of connections it has with other components. Degree centrality illustrates how many potential disruptions could occur if the component of interest was disrupted. Meanwhile, betweenness is a measurement of how many shortest paths would be disrupted were a specific component to fail. Stormwater runoff had the highest degree of centrality and betweenness, followed by infiltration, water body, water infrastructure, and power infrastructure. The degree and betweenness values for all system components are visualized in Fig. 3.

### Pathways of disruption

**Fig. 4:**
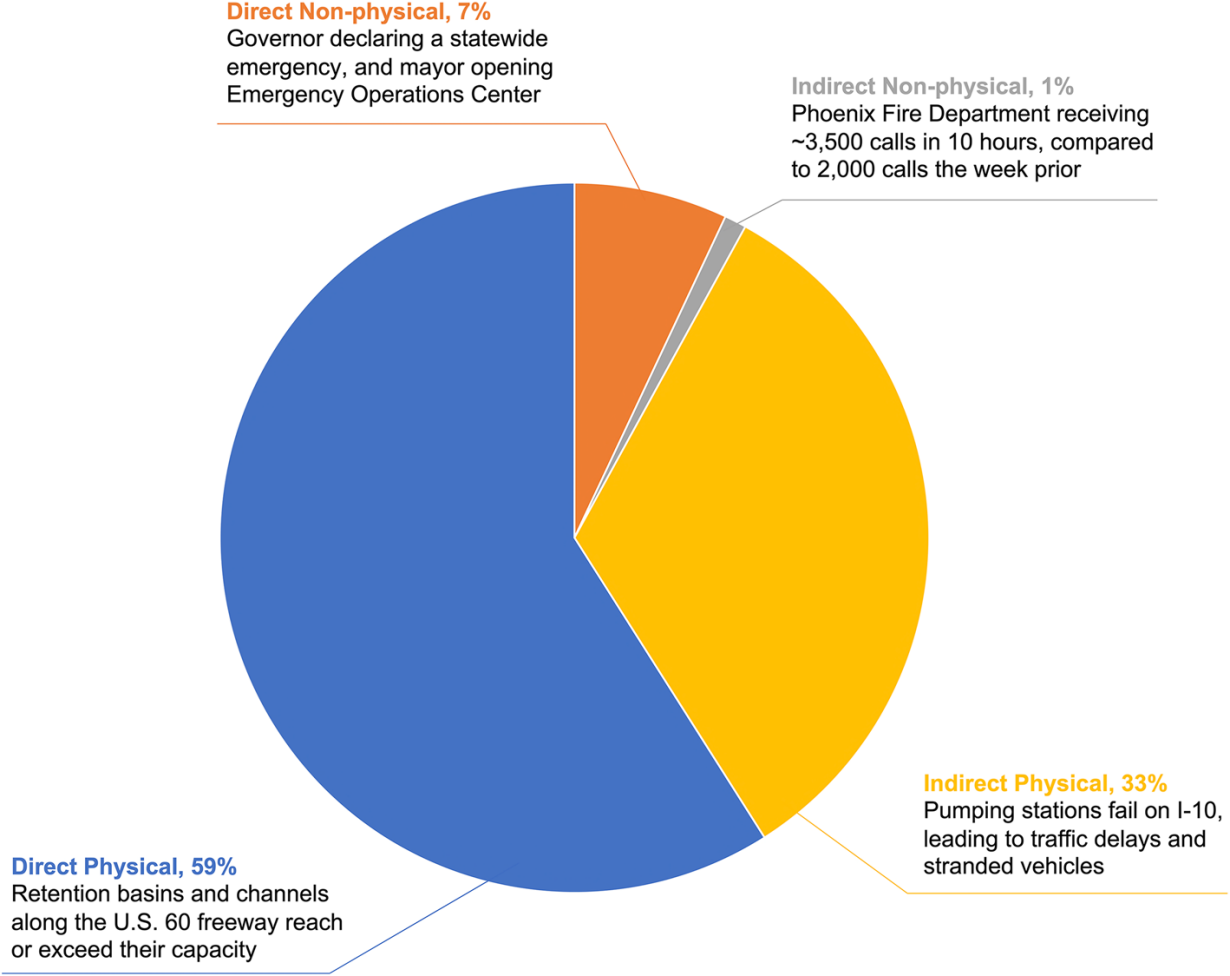
Occurrence of pathways of disruption classifications within the system relationships, along with an example of each classification

We first identified vulnerabilities to the urban system by verifying potential pathways of disruption between the social, ecological, and technological components, and we cataloged each relationship link within that pathway as either direct physical, indirect physical, direct non-physical, or indirect non-physical. 82 relationships were identified and consisted of 59% direct physical, 33% indirect physical, 7% direct non-physical, and 1% indirect non-physical (Fig. 4). The examples depicted in the figure, and elaborated here, are based on an actual extreme precipitation event on September 8, 2014. These examples are provided to emphasize that these pathways of disruption are already occurring and must be addressed in monsoon planning and response. During the event, retention basins and channels along the U.S. 60 freeway were close to exceeding capacity, demonstrating a direct physical pathway [57]. An indirect physical relationship developed when pumping stations failed on the Interstate-10 (I-10) causing traffic delays and stranded vehicles [57, 58]. Non-physical incidents included the declaration of a statewide emergency and the emergency response communications being overloaded, which were direct and indirect relationships, respectively [58, 59].

When assessing the 82 relationships, researchers and practitioner interviewees identified 10 significant pathways of disruption. All identified pathways begin with stormwater runoff disrupting CI. This included direct impacts on water, power, transportation, petroleum, and gas infrastructures. (Stormwater runoff could also directly impact residential and non-residential infrastructures; however, there were no indirect impacts thereafter.) Of these, power infrastructure appears to be the highly critical based on centrality calculations and interviews, due to the greatest potential for cascading failure to other sectors, including transportation, water, gray stormwater, gas, petroleum, ICT, residential, and non-residential infrastructures. Power infrastructure is tightly coupled with water infrastructure, indicating another potential pathway of disruption. The disruption of power and gas services can cause residential and non-residential infrastructures to have limited or degraded services. This is extremely critical to human health, as the monsoon season coincides with the hottest time of the year when individuals need access to potable water and cooling. Citizens also rely upon the Flood Control District of Maricopa County’s website and local news reports for up-to-date precipitation reports and maps during extreme weather events [59], access to which requires power services.

Transportation infrastructure is vulnerable to disruption directly from stormwater runoff, but also indirectly through failures of power and water infrastructures, such as a power outage leading to a stop light becoming a four-way stop or failed pumping leading to inaccessible roadways due to flooding. When roadways become inundated, individuals may not be able to access residential and non-residential infrastructure (e.g., schools, hospitals). Non-residential infrastructure are defined as any CI not explicitly named in Fig. 2, such as businesses, manufacturing, health care, and education. Road closures could also disrupt emergency response teams (e.g., police, firefighters) and the delivery of goods (e.g., petroleum). Power outages can restrict emergency response teams by disrupting transportation infrastructure as well as communication lines.

### Significant feedback loops

**Fig. 5:**
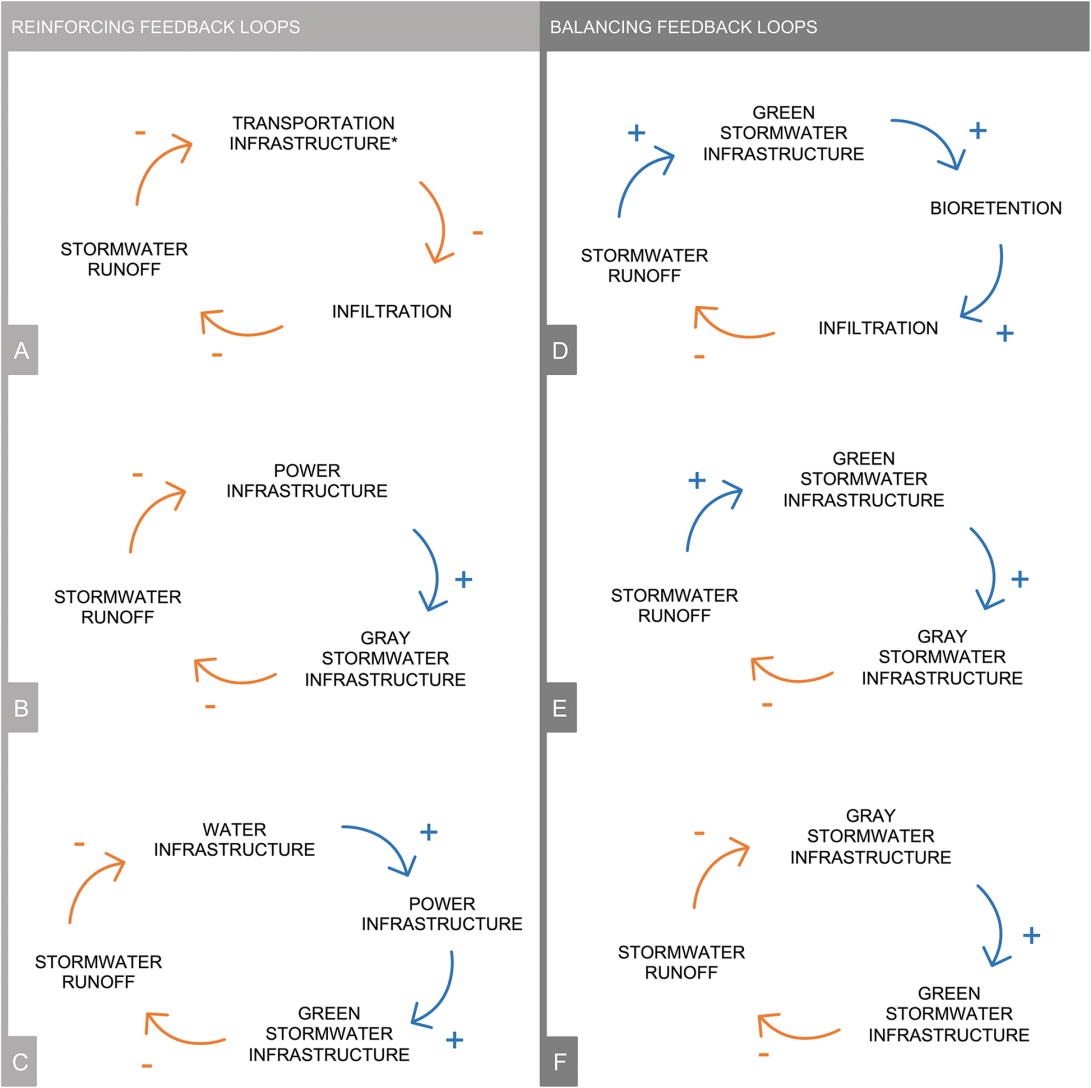
Significant reinforcing and balancing feedback loops, with flow categorization (positive ( +), negative (-)) based on flooding consequences. *Transportation infrastructure could be substituted for gray stormwater infrastructure, green stormwater infrastructure, residential buildings, and non-residential buildings

The second approach for discovering vulnerabilities within the urban system was to identify feedback loops present among the relationships between social, ecological, and technological components. Feedback loops can be either balancing (i.e., cycle stabilizes over time) or reinforcing (i.e., cycle results in growth or decline) [60]. In the context of flooding, a balancing loop would aid in maintaining service of and accessibility to the technological components, supporting ecological processes, and preventing negative social impacts. Meanwhile, a reinforcing loop would perpetuate flooding risks across the SETS domains. The ecological processes (e.g., infiltration, bioretention, evapotranspiration) play an important role in creating feedback loops, emphasizing the significance of the water cycle in urban flood dynamics. Urban development modifies the water cycle by reducing water storage capacity, altering infiltration rates, changing precipitation patterns, etc. [61, 62], this development can hinder ecological processes, causing less effective stormwater management. For example, impervious surfaces limit on-site infiltration, leading to increased stormwater runoff [63], this feedback loop is depicted in Fig. 5A. However, integrating more green infrastructure alongside gray infrastructure can help reduce stormwater runoff, as demonstrated by the balancing loops in Fig. 5D, E, and F. The implementation of more bioretention through green infrastructure can reduce flood consequences (Fig. 5D), and this flood management strategy was frequently mentioned by practitioner interviewees, who also pointed to the potential co-benefits (e.g., recreational space). Figure 5E and F demonstrate how green and gray infrastructures can complement each other to reduce the strain placed on either system, providing an opportunity for redundancy. Finally, within the CI components, power infrastructure is a clear point of vulnerability due to the number of components that rely upon it (Fig. 5B). Due to the tightly coupled relationship between water infrastructure and power infrastructure, water infrastructure additionally becomes a critical component (Fig. 5C).

## Discussion

The illustrative causal loop diagram demonstrates how social, ecological, and technological domains are impacted by a monsoon event in Phoenix, and how relationships between the three domains interact and affect each other, either amplifying or reducing the impact of the event. The monsoon event, which is represented by the ‘Precipitation’ and ‘Stormwater Runoff’ components (highlighted red in Fig. 1), has the most potential for causing direct and indirect physical disruptions to water and power infrastructures. The majority of this interaction is driven by stormwater runoff, which is the component with the highest degree and betweenness centrality. While degree and betweenness report the same top five components for centrality, emergency response and ICT infrastructure (social-technological components) emerge as potential bottlenecks (as reported by betweenness). This emphasizes the need to examine complex systems from a variety of perspectives. Interviewed flood managers also highlighted the importance of effective emergency response for mitigating the 12 social flood impacts identified in the SETS causal diagram. Interviewees additionally stressed the importance of building capacities for effective response at both the institutional (e.g., adequate planning, operation during the event, funding and resource allocation, infrastructure serviceability) and community (e.g., access to information and infrastructure services, household preparedness) levels. Stormwater infrastructures are a significant component in controlling various hydrological risks as emphasized in Fig. 4. However, once stormwater infrastructures are overwhelmed, they may be less effective in controlling the cascading effects, particularly in gray infrastructure systems. This emphasizes the need for technological infrastructure to be responsive to changing climate conditions (e.g., robustness, adaptation, transformation) to limit cascading disruptions. The network analysis reaffirms the usefulness of a SETS perspective for monsoon planning and response as the interactions between social, ecological, and technological system components in the illustrative causal diagram reveal several potential pathways of disruption.

### Applying SETS to identify pathways of disruption

The problem of pluvial flooding is occasionally overlooked because it is considered ‘solved’ by technological stormwater infrastructure [64]. However, interactions between social, ecological, and technological components contribute to the magnitude of disturbance experienced during extreme precipitation events. The inclusion of the water cycle resulted in a large redundancy within feedback loops, as the cycle provided multiple movement pathways for water (e.g., infiltration, water storage, evapotranspiration). Urban development disrupts the natural water cycle, influencing hydrologic stores and fluxes [65, 66] and reduces the effectiveness of stormwater infrastructure, leading to flooding, when improperly managed. For example, the large amounts of impervious surfaces in Phoenix can lead to quickly overwhelmed drainage systems during flash flooding [50, 67, 68]. Impacts of flooding vary spatially across the city, with poor or marginalized communities often experiencing greater burdens of pluvial flooding due to inadequate infrastructure [68, 69].

Academic literature on extreme precipitation events in Phoenix tends to focus more on direct physical pathways of disruption compared to indirect physical, direct non-physical, and indirect non-physical disruptions (Supplementary Information Table 1), corroborating findings from [17]. Gray literature (e.g., news articles), on the other hand, does commonly inform audiences of all impacts: commutes (e.g., flooded residential streets), damages (e.g., infrastructure failures), dangers (e.g., power outages), etc. The oversight of disruptions beyond direct physical disruptions in literature can lead to unexpected consequences and examining *pathways* of disruption provides insights toward increasingly interconnected and interdependent CI systems. The identified indirect physical disruptions were most likely to occur between infrastructures. Therefore, as CI systems become more interconnected and interdependent, it is important to consider symbiotic relationships, not only in times of operation but in times of disruption. In other words, infrastructure managers must consider how the failure of one infrastructure may ripple across the urban system. Further, we have only examined one disturbance: an extreme precipitation event. In Phoenix, extreme precipitation often coincides with extreme heat. Concurrent hazards provide a heightened threat to infrastructure systems, indicating how climate change may overwhelm existing CI [5].

The analysis of potential pathways of disruption within an urban system–as related to an abstract, severe extreme weather event–indicates numerous instances where disruption can extend beyond a single component that may be the focus of management (e.g., stormwater infrastructure associated with transportation). Sequences of potential cascading failures demonstrate opportunities for symbiotic relationships between and within social, ecological, and technological components to prevent or prepare for subsequent disruptions. Feedback loops within the urban system can either enhance or deteriorate efforts of symbiotic relationships between components, so awareness of these relationships and impacts can help shape development and policies. For instance, the City of Phoenix requires that post-development peak discharges do not exceed pre-development peak discharges for 2-, 10-, and 100-year (recurrence interval) storms and further requires retention and/or treatment of the ‘first flush,’ the first 0.5 inches of direct stormwater runoff from a precipitation event [70, 71]. These policies were put in place to minimize pollutant releases, which can be reduced by green infrastructure, such as multifunctional natural drainage corridors (e.g., Indian Bend Wash) or rainwater collection [71, 72]. Ideally, these initiatives of integrating gray and green infrastructures to manage stormwater could become standardized for retrofitting existing developments as well [71]. In short, while pathways of disruption highlight potential weak spots within the system that would benefit from climate adaptation (e.g., consequence-based management, strengthening infrastructure, multifunctionality), feedback loops may serve as potential tools to divert failure at the root cause.

### Recommendations for holistic monsoon planning and response

The examination of cascading failures during an extreme precipitation event provides an opportunity for flood managers to explore design consequences from a holistic perspective, but also requires explicit acknowledgement of the complexity surrounding SETS. One participant summarized the monsoon season as “predictably unpredictable.” The interviews revealed four themes that can enhance holistic monsoon planning and response: collaboration (7 out of 8 participants), innovation (6), education (4), and equity (4). The opportunities emphasize that social, ecological, and technological components are needed to comprehensively address pluvial flooding, and technology will only be one piece of the puzzle toward fostering increasingly sustainable and resilient communities.

The participants were adamant about developing a community of stakeholders involved in monsoon planning and response. The community of stakeholders would meet at regular intervals to create joint preventive strategies. The participants emphasized the need for preventive planning, as systems are already in place for reactive response. This *collaboration* would provide opportunities for cross-jurisdictional (e.g., community to county scale) and cross-sectoral (e.g., engineering and landscape architecture) learning and development of shared visions. A shared vision could be particularly valuable as stakeholders could then combine resources to effect larger-scale change. Finally, two participants also discussed how such collaboration could facilitate systems thinking by providing a space for diverse perspectives to emerge and long-term strategies to be discussed. It was also noted by a participant that while the SETS framework is useful for facilitating systems thinking; however, it is not well-known nor particularly accessible.

While discussed in a number of contexts, there was recurring support for more formal governance to support *innovative* stormwater management. Participants wished to see policy advocacy (and, consequently, funding) for long-term and regional planning and policy support for alternative stormwater management strategies, such as green infrastructure. However, they noted that the state of Arizona is generally conservative and “cities are apprehensive to risk,” which leads to many innovative efforts being voluntary (across industry, commercial, municipal, and residential operations). To encourage voluntary efforts by reducing the learning curve, participants suggested developing manuals, such as the LID Toolkit for Mesa (2015), LID Toolkit for Glendale (2015), and Greater Phoenix Metro Green Infrastructure Handbook (2019).

The final two themes emphasize community engagement through *education* and *equity*. A significant concern for stakeholders is knowing where there is (or will be) flooding and communicating potential consequences of the flooding (e.g., roadways as conveyance, evacuation procedures) or flood mitigation (e.g., green infrastructure maintenance). One participant was quoted saying, “Here, people forget about rain.” However, community education is bidirectional. Residents are experts on their communities and can provide insights on frequently flooded spaces. Community members must feel empowered to advocate for themselves or be actively recruited to engage in discussion. Half of the participants also recognized that disruptions would have varying degrees of impact depending on the socio-demographic factors of an affected community, emphasizing the need for equitable distribution of social, ecological, and technological resources to address flooding.

## Conclusion

Systems thinking — here, framing infrastructure(s) as social-ecological-technological systems) — could help reveal hidden complexities and better prepare CI (physical and institutional) to respond to growing uncertainty. We developed an illustrative causal loop diagram through literature review and semi-structured interviews with flood managers to visualize the complexity surrounding monsoon planning and response. The interviews also resulted in recommendations for collaboration, innovation, education, and equity to engage systems thinking in monsoon planning and response. This exercise in sensemaking allows flood managers to explore uncertainties and risks brought forth by interconnected social, ecological, and technological systems. This research provides a process for mapping interconnected, complex systems and integrating stakeholders in research, advancing knowledge in fields such as urban resilience, multisector dynamics, decision-making, and infrastructure futures. By considering diverse perspectives (social, ecological, and technological across infrastructure sectors), we can expand our collective knowledge and identify disturbances that were previously overlooked in planning and response, transforming unknown unknowns into known unknowns.

## Methods

We engaged in systems thinking through qualitative approaches: causal loop diagrams (CLD), literature review, and semi-structured interviews [60, 73–75]. CLDs serve various purposes, from promoting conversations among experts, testing hypotheses, developing research questions, identifying gaps in policy, and building theory [60, 76]. In the context of systems thinking, CLDs present a holistic view of a phenomenon instead of individual parts [77] that allows for further understanding of behavioral drivers in the system, including how elements influence each other [60, 74]. CLDs also reveal ‘leverage points’ in a system, which can become points of intervention in the policy arena [78]. Component influences can be visualized through direct (positive) and inverse (negative) relationships [60]. CLDs are deployed in this project to understand and build theories regarding current patterns of interaction between social, ecological, and technological systems during an extreme flooding event in Phoenix, AZ.

To validate the illustrative causal loop diagram and further evaluate the role of SETS in creating opportunities or challenges for resilience pathways in flood management, we conducted semi-structured interviews with key stakeholders in Phoenix across social, ecological, and technological systems. Semi-structured interviews are a qualitative research methodology aimed to explore participants' experiences, beliefs, and feelings on a specific topic [73]. In new areas of research, such as SETS, semi-structured interviews are suitable for a deep, topical understanding, including stakeholders' perceptions.

### Data collection and analysis

To create the CLD, the first and second authors worked in disciplinary silos on ecological and technological systems related to flooding. Following this, they presented their CLDs to the NSF Growing SETS Convergence project members (i.e., urban resilience experts). After iterative feedback and revisions, the ecological and technological systems diagrams were merged and the social components (composed mainly of institutions directly related to flooding in Phoenix) were integrated from data collected through literature and media. Following this integration, a recursive process was used to identify the interactions among social, ecological, and technological systems, focusing on flooding. All relationships were validated through literature and media review (i.e., academic articles, governmental documents, policy reports, and newspapers published from 2010 to 2020). Centrality was measured by degree and betweenness. Degree represents the number of relationships (i.e., adjacent edges) to other components from the component (i.e., node) of interest [79]. This measurement assumes that the most connections is equivalent to the most important. This measurement was selected to identify the potential number of disruptions possible if a component were to fail. Betweenness depicts the number of shortest paths in the system that use that component [79]. If a component has a high betweenness score, it is more likely to disrupt the system should it go offline because many other components are dependent upon its operation. The network analysis and centrality calculations were performed in RStudio Version 1.4.1717, using the tidyverse, igraph, and ggraph packages.

Select practitioner stakeholders were identified through convenience sampling for semi-structured interviews. Their roles spanned engineering, sustainability, emergency response, floodplain management, landscape architecture, environmental compliance across social, ecological, and technological sectors from municipal to county to state levels. The interview protocol consisted of open-ended questions about flooding impacts in Phoenix, SETS relationships, and institutional arrangements. Eight interviews were conducted by the two lead authors (one interviewer and one observer) via Zoom between April and June 2022, lasting between 35 and 45 min. The semi-structured interviews were analyzed via two methods. First, the diagram validation occurred through direct critique during the interview, providing instant feedback that was recorded by the observer. The diagram was not updated between interviews, so all participants critiqued the same diagram. Interviews were transcribed (via audio-to-text software) and reviewed to finalize diagram validation, when recording was permitted by the participant. Second, themes were derived via repetition from the interviews to summarize participant feedback regarding organizational and institutional learning as informed by systems thinking. The themes were developed and refined following Ryan and Bernard techniques [80].

### Supplementary Information

**Additional file 1: Supplementary Table 1.** Relationships between the components along with their link direction(s) and physical or non-physical classification. Direct relationships are defined as those resulting directly from precipitation or stormwater runoff, while indirect relationships were subsequent. This is not an exhaustive list.

## Data Availability

Interview data are not available due to privacy restrictions. Dataset generated from the gray literature review is presented in Supplementary Information.
