# Supplementary material for: Interdependence of social-ecological-technological systems in Phoenix, Arizona: consequences of an extreme precipitation event
Source: J Infrastruct Preserv Resil. 2023 Aug 18;4(1):19. doi: 10.1186/s43065-023-00085-6 (PMC10439024; doi:10.1186/s43065-023-00085-6)
Supplement: Supplementary file 1 — Additional file 1: Supplementary Table 1. Relationships between the components along with their link direction(s) and physical or non-physical classification. Direct relationships are defined as those resulting directly from precipitation or stormwater runoff, while indirect relationships were subsequent. This is not an exhaustive list. [file 43065_2023_85_MOESM1_ESM.docx]

**Supplementary Information**

Table 1. Relationships between the components along with their link direction(s) and physical or non-physical classification. Direct relationships are defined as those resulting directly from precipitation or stormwater runoff, while indirect relationships were subsequent. This is not an exhaustive list.

| **Relationships** | | **Link Direction** | **Classification** |
| --- | --- | --- | --- |
| *From* | *To* |  |  |
| **Precipitation** | Stormwater Runoff | Direct | Physical |
|  | Green Stormwater Infrastructure | Direct | Physical |
|  | Gray Stormwater Infrastructure | Direct | Physical |
|  | Bioretention | Direct | Physical |
|  | Infiltration | Direct | Physical |
|  | Rainwater Collection | Direct | Physical |
|  | Water Body | Direct  Inverse | Physical |
| **Stormwater Runoff** | Green Stormwater Infrastructure | Direct | Physical |
|  | Gray Stormwater Infrastructure | Direct | Physical |
|  | Bioretention | Direct | Physical |
|  | Infiltration | Direct | Physical |
|  | Evapotranspiration | Direct | Physical |
|  | Water Body | Inverse | Physical |
|  | Water Infrastructure | Inverse | Physical |
|  | Power Infrastructure | Inverse | Physical |
|  | Transportation Infrastructure | Inverse | Physical |
|  | Petroleum Infrastructure | Inverse | Physical |
|  | Gas Infrastructure | Inverse | Physical |
|  | Emergency Response | Direct | Non-physical |
|  | Residential Infrastructure | Inverse | Physical |
|  | Non-Residential Infrastructure | Inverse | Physical |
| **Green Stormwater Infrastructure** | Stormwater Runoff | Inverse | Physical |
|  | Gray Stormwater Infrastructure | Direct | Physical |
|  | Bioretention | Direct | Physical |
|  | Infiltration | Direct | Physical |
|  | Evapotranspiration | Direct | Physical |
|  | Water Body | Direct | Physical |
| **Gray Stormwater Infrastructure** | Stormwater Runoff | Inverse | Physical |
|  | Green Stormwater Infrastructure | Direct | Physical |
|  | Bioretention | Inverse | Physical |
|  | Infiltration | Inverse | Physical |
|  | Water Body | Direct  Inverse | Physical |
| **Bioretention** | Stormwater Runoff | Inverse | Physical |
|  | Infiltration | Direct | Physical |
|  | Evapotranspiration | Direct | Physical |
| **Infiltration** | Stormwater Runoff | Inverse | Physical |
|  | Bioretention | Inverse | Physical |
|  | Evapotranspiration | Inverse | Physical |
|  | Groundwater | Direct | Physical |
| **Evapotranspiration** | Stormwater Runoff | Inverse | Physical |
|  | Bioretention | Inverse | Physical |
|  | Infiltration | Inverse | Physical |
|  | Water Body | Direct | Physical |
| **Rainwater Collection** | Stormwater Runoff | Inverse | Physical |
|  | Infiltration | Direct | Physical |
|  | Water Infrastructure | Direct  Inverse | Physical |
| **Groundwater** | Water Body | Direct | Physical |
|  | Water Infrastructure | Direct | Physical |
| **Water Body** | Stormwater Runoff | Direct | Physical |
|  | Evapotranspiration | Direct | Physical |
|  | Groundwater | Direct | Physical |
|  | Water Infrastructure | Direct | Physical |
| **Water Infrastructure** | Groundwater | Inverse | Physical |
|  | Water Body | Inverse | Physical |
|  | Power Infrastructure | Direct | Physical |
|  | Petroleum Infrastructure | Direct | Physical |
|  | Residential Infrastructure | Direct | Physical |
|  | Non-Residential Infrastructure | Direct | Physical |
| **Power Infrastructure** | Gray Stormwater Infrastructure | Direct | Physical |
|  | Water Body | Inverse | Physical |
|  | Water Infrastructure | Direct | Physical |
|  | Transportation Infrastructure | Direct | Physical |
|  | Petroleum Infrastructure | Direct | Physical |
|  | Gas Infrastructure | Direct | Physical |
|  | ICT Infrastructure | Direct | Non-physical |
|  | Residential Infrastructure | Direct | Physical |
|  | Non-Residential Infrastructure | Direct | Physical |
| **Transportation Infrastructure** | Infiltration | Inverse | Physical |
|  | Water Body | Direct | Physical |
|  | Petroleum Infrastructure | Direct | Physical |
|  | Emergency Response | Direct | Non-physical |
|  | Residential Infrastructure | Direct | Non-physical |
|  | Non-Residential Infrastructure | Direct | Non-physical |
| **Petroleum Infrastructure** | Groundwater | Direct | Physical |
|  | Transportation Infrastructure | Direct | Physical |
| **Gas Infrastructure** | Groundwater | Inverse | Physical |
|  | Residential Infrastructure | Direct | Physical |
|  | Non-Residential Infrastructure | Direct | Physical |
| **ICT Infrastructure** | Emergency Response | Direct | Non-physical |
| **Emergency Response** | ICT Infrastructure | Inverse | Non-physical |
| **Residential Infrastructure** | Infiltration | Inverse | Physical |
| **Non-Residential Infrastructure** | Infiltration | Inverse | Physical |
